# Can health technologies be “care optimizers”? A normative evaluation of digital health technologies in light of postphenomenological reflections

**DOI:** 10.1007/s11019-026-10337-3

**Published:** 2026-03-05

**Authors:** Orhan Onder, Ilhan Ilkilic

**Affiliations:** 1 Department of History of Medicine and Ethics, School of Medicine, Marmara University, Başıbüyük Yolu No: 9 D:2, 34854 Maltepe, Istanbul Turkey; 2 Chair for Philosophy of Media and Technology, University of Vienna, Universitätsstrasse 7, 1010 Vienna, Austria; 3 Department of History of Medicine and Ethics, Istanbul Faculty of Medicine, Istanbul University, Eski Esnaf Hastanesi, 34116 Fatih, Istanbul Turkey

**Keywords:** Technological mediation, Clinical decision support systems (CDSS), Postphenomenology, Care optimizer, Doctor–patient relationship, Health technologies

## Abstract

The integration of new technologies such as artificial intelligence (AI) into healthcare has initiated profound changes in clinical practice, reshaping not only clinical workflows but the relational structure of care itself. Among the most significant developments is the transformation of the traditional doctor–patient dyad into a doctor–patient–technology triad. Philosophical studies of technology, especially postphenomenological research, argue that technologies should not be understood as neutral tools but as mediators of perception and action. Building on this perspective, we argue that ethical evaluation must move beyond description toward a normative orientation for understanding how such mediation reshapes care relations. To this end, we draw on postphenomenological theory of technological mediation in relation to contemporary clinical practices and develop a normative evaluative framework grounded in patient wellbeing, understood as an emergent, relational quality of care. Rather than deriving evaluative categories from a single tradition, the framework integrates insights from quality-of-care scholarship and care-ethical perspectives, informed by relational accounts of clinical practice. It operationalizes this orientation across five domains (care quality, patient-centeredness, decision-making, access and communication, and care practices) understood as relational sites where technological mediation can enhance or compromise care. Using clinical decision support systems (CDSS) as an analytically demanding test case, we introduce the concept of the *care optimizer* as a descriptive-normative category identifying technologies that structure the conditions under which care unfolds. By anchoring evaluation in patient wellbeing and situating technological mediation within a normative horizon, the framework translates postphenomenological insights into ethically actionable guidance and aims to ensure that digital health innovations reinforce, rather than undermine, the moral commitments of medicine.

## Introduction

The integration of digital health technologies has fundamentally reshaped the architecture of care (Nassehi et al. 2024). Where medical practice once revolved around a doctor–patient dyad, contemporary healthcare increasingly operates in triadic configurations in which technological systems co-structure clinical encounters. Computer-mediated practices, exemplified by Clinical Decision Support Systems (CDSS), AI-based diagnostic tools, and patient platforms, are now central to medical visits (Marino et al. 2023).

Triadic arrangements are not entirely new: family members, nurses, and consultants have long mediated doctor–patient interactions (Laidsaar-Powell et al. 2013; Bar-Haim 2018; McDowell 1962). Yet health technologies differ categorically from human intermediaries. They do not “provide” care or occupy a moral subject position but mediate it—structuring perception, decision-making, and communication (Antes et al. 2021). Despite this transformative role, technologies are still often conceptualized as neutral tools, serving human intention to improve accuracy or efficiency. Postphenomenological critique challenges this instrumentalist view, emphasizing that technologies actively shape human perception and action (Verbeek 2005). They condition how symptoms are interpreted, risks assessed, and responsibilities distributed across human and non-human actors (Verbeek 2011; Coeckelbergh 2020). Technologies thus mediate moral subjectivity and clinical judgment, influencing what counts as evidence, who is credible, and which options become visible or obscured (Verbeek 2007).

Drawing on postphenomenology, we argue that digital health systems mediate not only clinical information but also the relational and moral dimensions of care. Postphenomenology offers a relational ontology that captures how technologies co-constitute human experience—filtering perception and configuring possibilities for action—rather than merely extending capacity (Verbeek 2016; Ihde 1990). In this light, technologies such as CDSS cannot be understood as passive aids; they are moral actors that shape goals, values, and quality of care.

This article addresses the question: *How should health technologies be conceived as mediators within the evolving relational practices of care so that patient wellbeing is meaningfully supported?* Moving beyond neutrality, we conceptualize these systems as technological mediators that structure decision-making and clinical interaction. Within this perspective, we introduce the notion of the care optimizer—a descriptive and normative category that highlights how technologies condition the unfolding of care and redistribute responsibility without anthropomorphizing them (Verbeek 2008; Kroes and Verbeek, 2014). Care optimizers are neither providers nor receivers but actors that mediate the conditions under which care is experienced, structured, and ethically evaluated.

The paper has two aims. First, it offers a conceptual account of health technologies as mediators, challenging instrumentalist assumptions that dominate biomedical discourse. Second, it develops a normative basis for assessing technological mediation by positioning patient wellbeing as the ethical criterion for care optimization. This criterion grounds five domains—care quality, patient-centeredness, decision-making, access and communication, and care practices—that serve as relational sites for analyzing how technologies shape clinical life.

As Hansson and Fröding (2024) note, digital tools in healthcare raise distinctive ethical challenges concerning human contact, patient wellbeing, and justice. In an era where clinical lifeworlds are increasingly mediated by algorithms and platforms, ethical reflection on these mediations is no longer optional. By framing technologies as care optimizers and grounding their evaluation in patient wellbeing, this article offers a conceptual and normative orientation for ensuring that digital health innovations reinforce—not erode—the moral fabric of care.

The argument proceeds as follows: Sect. "From dyads to triads: the changing topology of care relations" traces the shift from dyads to triads and explains why technological triads differ from traditional human-centered models. Section "Technological mediation: a postphenomenological framework" outlines the postphenomenological framework of technological mediation. Section "Technologies as care optimizers: a normative framework" introduces care optimization as a normative ideal, detailing the five domains through which technologies can enhance or compromise care. Section "Discussion" discusses ethical tensions, implications for clinical practice and governance, and limitations of the framework, followed by a conclusion on aligning digital health innovation with medicine’s moral commitments.

## From dyads to triads: the changing topology of care relations

The clinical encounter has traditionally been framed as a dyadic relationship between doctor and patient—an interaction structured around asymmetries of knowledge, authority, and responsibility. Today, healthcare rarely occurs within such a closed system. It is increasingly common for third entities—ranging from human intermediaries such as nurses and relatives, with distinctive dynamics of memory, communication, and agency (Johnsson et al. 2019), to non-human actors such as algorithms and software systems, now conceptualized as active members of diagnostic teams (James et al. 2023). This shift reflects the distributed nature of agency in modern clinical settings and has spurred growing interest in triadic relations (Laidsaar-Powell et al. 2013).

Yet not all triads are equivalent. Traditional models involving human agents, long studied in medical sociology and clinical communication, often preserve an underlying care receiver–provider dyad. In what follows, we first review these human-centered triads before contrasting them with technological triads, where decision-support systems and similar technologies fundamentally alter the topology of care.

### Traditional triads in care

The most widely studied triadic configurations involve a patient relative or caregiver alongside the physician and patient, especially in pediatric, geriatric, and palliative care, where patient autonomy may be limited. For example, Stubbe (2017) highlights how dementia patients, family members, and physicians negotiate agency and understanding, while Coe and Prendergast (1985) analyze alignment—and conflict—of goals among elderly patients, caregivers, and clinicians.

Another well-documented configuration involves nurses as the third party. McDowell (1962) conceptualized the doctor–nurse–patient triad as a homeostatic system, while Brink (1972) emphasized the nurse’s interpretive role in bridging medical authority and patient needs. Further studies confirm that patients, relatives, and nurses strive to establish care relationships through triadic encounters that navigate communication, agency, and shared understanding (Johnsson et al. 2019). Despite their structural complexity, these triads remain functionally dyadic: clinicians deliver care, and patients receive it.

Even specialized contexts, such as psychiatry, follow this logic. Bar-Haim (2018) describes triads where a psychiatrist joins an existing patient–psychotherapist dyad, yet care remains coordinated within a human-driven relational framework. These examples show that traditional triads rarely disrupt the binary of care receiver and provider; they complicate rather than transform its structure.

Before turning to technological triads, it is crucial to clarify that the “doctor–patient–technology triad” is not an a priori structural assumption, nor a geometric model that presupposes three fixed roles. Rather, the triad is a descriptive name for a relational configuration that has emerged through the historical integration of digital and AI-driven systems into clinical practice. In a postphenomenological sense, clinicians, patients, and technologies do not exist as stable entities prior to their relations; they are co-constituted through mediated practices of seeing, interpreting, and acting. The triad therefore marks a qualitatively different transformation in the ontology of care—one in which technologies reshape not only how diagnoses are reached or decisions are made, but also who the knower is, what kinds of knowledge count, and how moral agency is distributed. Thus, the triad is best understood as a surface heuristic that highlights an emergent focal point within a broader network of continuously shifting mediations, rather than a static structure added to the traditional dyad.

### The doctor–patient–technology triad

The rise of health technologies—from electronic health records (EHRs) and telemedicine to AI-enabled CDSS—has created a qualitatively different triadic configuration that cannot be mapped onto the care receiver–provider axis. Unlike human intermediaries, technologies lack moral agency and intentionality but are far from passive. They mediate, structure, and sometimes substitute for clinical reasoning, communication, and moral deliberation. Digital systems redefine the contours of truth and trust in clinical practice (Danaher and Sætra 2022). Despite their promise, AI tools must address issues like bias, justice, and explicability to ethically support clinical decision-making (Benzinger et al. 2023).

Consider the introduction of computers into examination rooms, which Scott and Purves (1996) found to reshape conversational flow and spatial dynamics. Similarly, EHR interfaces now actively structure what can be recorded, retrieved, or prioritized (Córdova González 2022). Patients enter consultations influenced by algorithmically filtered information—a phenomenon Freckelton (2020) calls “Dr. Google”. Telemedicine collapses physical co-presence while amplifying digital mediation, and AI-based CDSS extend this further by generating diagnostic hypotheses, risk scores, and treatment suggestions, often through opaque models (James et al. 2023; Triberti et al. 2020).

In these cases, technologies are neither patients nor clinicians, yet they reshape the moral, epistemic, and relational landscape of care. They introduce new asymmetries between designers and users, shift loci of expertise, and destabilize traditional patterns of trust, authority, and responsibility (Onder 2025). AI systems, for example, can reconfigure autonomy and confidentiality—effects requiring sustained ethical scrutiny (Ilkilic, 2020) and attention to how AI alters trust, reliability, and epistemic authority in care (Buhr et al. 2025). These technological triads demand conceptual and normative attention: if technologies reshape the conditions of care, do they enhance or undermine patient wellbeing?

We propose viewing technologies as a third focal entity—participants that cannot be reduced to providers or receivers but that shape how care is interpreted and enacted. This model reflects a transformation in the identities, epistemic roles, and agencies of all involved, rather than an additive extension of the traditional dyad. Drawing on postphenomenology and care ethics, we emphasize that clinicians, patients, and technologies emerge as moral and epistemic actors through their relations, not prior to them. The “triad” is therefore a heuristic that marks a new focal point within a dynamic field of mediations, rather than a geometric arrangement of three stable entities. Key questions follow: What role should technologies play in care? Are they accountable? Can they foster the conditions of care without compromising trust, autonomy, or empathy? Such issues cannot be addressed within an instrumentalist framework that treats technologies as neutral extensions of clinical intention.

To address these questions, we adopt a postphenomenological perspective that conceptualizes technologies as mediators of perception and action. This enables us to move beyond the “tool” metaphor and articulate technologies as participants in care—entities that may condition how care is delivered, understood, and valued. The next section develops this claim using the theoretical resources of technological mediation. In doing so, we shift from describing the structure of the technological triad to examining the mechanisms of mediation through which it reconfigures clinical reasoning and care practices.

## Technological mediation: a postphenomenological framework

The conceptualization of health technologies as potential care optimizers requires moving beyond the instrumentalist view of technology. In dominant biomedical discourse and health technology assessment, technologies are often seen as value-neutral tools (Ten Have 2004)—applied to achieve predefined clinical goals such as accuracy, efficiency, or risk reduction. This view subordinates technologies to human intention and overlooks their active role in shaping how care is understood and practiced. As the previous section indicated, health technologies now structure not only what is done in the clinic but also how care is perceived, communicated, and experienced.

As clarified in the previous section, technological mediation is not simply an influence on clinical interaction but the very mechanism through which the identities and capacities of “doctor,” “patient,” and “technology” become what they are within the relation. Postphenomenology holds that actors do not precede their relations: they emerge through webs of mediated practices. Thus, mediation is the process by which clinicians’ epistemic agency is reshaped, patients’ moral subjectivity is configured, and technologies become participants in care rather than neutral tools. Section "Technological mediation: a postphenomenological framework" therefore provides the conceptual machinery that explains the relational reconfiguration described in Sect. "From dyads to triads: the changing topology of care relations".

To capture this transformative role, we draw on postphenomenological accounts of technological mediation. Rooted in the works of Don Ihde and Peter-Paul Verbeek, postphenomenology offers a relational ontology that foregrounds how technologies co-constitute human perception, action, moral experience, and even moral and epistemic subjectivity—shaping what kinds of knowers clinicians can be, how patients appear as moral subjects, and how care itself becomes intelligible (Verbeek 2016). Postphenomenology rejects an additive view where technologies enter an otherwise stable dyad. Instead, technological mediation reconfigures the relations through which “doctor,” “patient,” and even “care” become meaningful roles. The triad is therefore not an expanded dyad but a qualitatively different relational ecology of co-constitution. Technologies such as CDSS are not merely adjuncts to clinical reasoning but participants in constructing clinical reality itself (Pols 2012).

Mediation theory thus explains not only how technologies influence clinical practice, but how they constitute the very subjects who participate in that practice. For clinicians, AI-enabled systems mediate what is salient, which risks become thinkable, which evidence is authoritative, and what forms of clinical judgment are permissible. For patients, digital interfaces, triage systems, and algorithmically curated information shape how vulnerability, responsibility, and agency are experienced. Technologies therefore participate in forming the moral topography of care—they help decide who can act, who must justify, and whose perspective becomes legitimate.

### From instrument to mediator

The shift from understanding technologies as instruments to viewing them as mediators is central to postphenomenology. Verbeek (2005) critiques the instrumentalist paradigm for failing to capture the ways technologies intervene in the human–world relation. Rather than external aids, technologies are integral to how experience is structured (Rosenberger and Verbeek 2015).

Ihde emphasizes the multistability of artifacts—the idea that a single technology takes on different functions depending on context (Ihde 1990). A stethoscope, for instance, can operate as a diagnostic tool, a symbol of authority, or a mediator of intimacy (Rosenberger 2012). He further develops the notion of **e**mbodiment relations, where technologies become incorporated into the user’s sensory horizon. Through a microscope, the world is not simply viewed differently—it is constituted differently, altering both perception and the questions posed (Ihde 1990). Building on this, Verbeek (2005, 2011) argues that technologies also mediate human action by possessing a form of *technological intentionality*. While they lack consciousness or goals, they shape the direction of human intentions. A decision-support system that presents some treatment options while excluding others frames clinical judgment, guiding choices in particular directions.

Verbeek’s concept of “material hermeneutics” deepens this point: technologies do not merely provide information but interpret the world for us. In clinical contexts, CDSS and diagnostic interfaces establish hermeneutic frameworks that shape how clinicians read bodily signs, how explanations are constructed, and what counts as a valid inference. These mediating scripts recalibrate the clinician’s epistemic agency by prefiguring avenues of reasoning, altering evidential weight, and structuring the horizon of possible diagnoses. Material hermeneutics thus bridges the relational ontology of Sect. "From dyads to triads: the changing topology of care relations" with the concrete mechanisms through which technologies participate in clinical reasoning.

Within this postphenomenological framework, technological mediation can be analytically distinguished into two closely related processes: the mediation of perception and the mediation of action (Hauser et al. 2018).

Mediation of perception refers to how technologies filter and reframe sensory access to the world. Ultrasound imaging, for example, not only “reveals” the fetus but constructs it as a patient, a life, or a moral subject, transforming parental and clinical decisions (Verbeek 2011). Similarly, CDSS mediate perception by structuring which data are flagged, how risks are visualized, and what counts as clinically relevant.

Mediation of action denotes how technologies configure possibilities for intervention. EHR systems that require specific data entry before prescribing influence the sequence and scope of decision-making (Moerenhout et al. 2020). Predictive analytics embedded in CDSS may suggest predefined treatment paths, effectively scripting clinical responses. These mediations are not deterministic—postphenomenology resists technological determinism—but they illustrate how design and context shape human–technology interaction.

### Ethical implications and technologies as moral actors

Understanding mediation as both perceptual and practical has direct ethical implications. If technologies shape what is perceptible and actionable, they likewise mediate what care demands in a given moment—what forms of attentiveness become possible, whose vulnerabilities are acknowledged, and how responsibilities are distributed. Traditional responsibility frameworks and explainability requirements that assume discrete human agents are challenged when decision-making is mediated by opaque algorithms (Funer et al. 2024a; Gordijn and Ten Have 2023). Questions of explainability and accountability arise: if clinicians rely on CDSS, can they remain fully accountable for decisions (Funer et al. 2024b)?

Verbeek (2011) argues that technologies participate in the moral composition of action by structuring the field of possibilities. CDSS may highlight certain risks while rendering others invisible, directing attention in ethically significant ways. This suggests that the agency is distributed across networks of human and non-human actors—physicians, algorithms, data infrastructures, and protocols (Coeckelbergh 2012, 2020). Responsibility, therefore, must be understood relationally and situatedly (Onder 2025).

Technologies also shape moral subjectivity—how individuals understand themselves and their responsibilities. These mediations also generate new asymmetries and dependencies. Algorithmic systems can amplify epistemic asymmetries by privileging certain forms of data-driven evidence over experiential or narrative knowledge (Onder 2022, 2025). Moreover, the opacity of AI models creates novel vulnerabilities for clinicians and patients, who must rely on recommendations they cannot always interpret or contest. Care ethics has long emphasized relations of dependence, responsiveness, and vulnerability; technological mediation restructures these relations, shifting who depends on whom, and on what grounds. In this sense, mediation is not only epistemic and practical but inherently ethical, as it redistributes responsibility, exposure, and relational power. Self-tracking devices, for instance, encourage patients to view themselves as data producers accountable for health management, shifting responsibility from professionals to individuals (Boer and Kudina 2021). Such shifts affect autonomy, dependency, and trust within clinical relationships.

Verbeek’s notion of moralizing technology (2011) captures this dynamic. Technologies are not moral agents but moral actors: they guide, constrain, and enable moral action. The ranking of CDSS treatment options, for example, encodes implicit value judgments about risk, desirable outcomes, and evidentiary standards. These embedded assumptions highlight the ethics of design as much as the ethics of use (Verbeek 2016).

This postphenomenological stance blurs ontological and normative boundaries. Technologies are not neutral objects but relational artifacts that co-construct moral meaning. This view echoes broader trends in Science and Technology Studies (STS) and new materialist ethics, which emphasize the entanglement of materiality and normativity (Morrison 2020). In healthcare, this means CDSS, telemedicine platforms, and AI-driven diagnostic tools not only mediate decisions but reshape the terrain of moral deliberation itself—defining what is visible, actionable, and valuable (Kudina 2021; De Boer and Hoek 2020).

Recognizing technologies as moral actors thus enables a more responsible approach to their integration. It also sets the stage for normative frameworks that evaluate technologies not only by technical performance but also by their contribution to moral considerations and patient wellbeing. Technological mediation is therefore the mechanism through which the triadic relational topology described in Sect. "From dyads to triads: the changing topology of care relations" is continually constituted and reconstituted. Through mediation, technologies do not merely influence care—they participate in shaping the identities, capacities, and moral relations of every actor involved.

## Technologies as care optimizers: a normative framework

As healthcare technologies increasingly shape clinical practice, their role must be understood not only in technical or instrumental terms but also within a normative framework. Postphenomenology shows that technologies mediate perception, action, and moral meaning; however, this descriptive insight leaves open what ethical ends such mediation should serve. To address this, we introduce the concept of the care optimizer—*a normative and conceptual category for evaluating how technologies should co-shape care*. Framing technologies such as CDSS as care optimizers highlights their morally consequential role in structuring clinical encounters and provides a lens for assessing their contribution.

The aim of this framework is not to measure or optimize care in a managerial sense, but to articulate the relational conditions through which *good care* can emerge in technologically mediated settings. Rather than treating wellbeing as an outcome to be achieved, we understand it as an unfolding, co-constructed quality of clinical encounters that arises through attentiveness, responsiveness, trust, and shared interpretation. The five domains therefore identify key relational practices—moments in which technologies can support or unsettle the fragile dynamics of caring relations. In line with the postphenomenological account developed in Sect. "Technological mediation: a postphenomenological framework", the framework therefore evaluates technologies not by discrete outputs, but by how they reconfigure the epistemic, moral, and relational conditions under which care becomes possible.

Care ethics complements this approach by emphasizing attentiveness, responsiveness, and relational integrity as central values of good care. These values orient technological mediation toward the ethical telos of medicine: the protection and promotion of patient wellbeing. In this sense, the *care optimizer* functions as an interpretive lens and a relational orientation point for asking whether technological mediation sustains, distorts, or redistributes the moral fabric of clinical practice.

Although CDSS function in this article as a primary empirical reference point, they are not treated as merely one example among many digital health technologies. They are selected because they represent one of the most ethically consequential and epistemically interventionist forms of technological mediation currently embedded in clinical practice. Unlike peripheral or administrative tools, CDSS directly shape diagnostic reasoning, responsibility distribution, and value-laden decision frames. For this reason, they provide a particularly demanding test environment for the care optimizer framework: if the normative criteria developed here remain meaningful under the complex and high-stakes mediation characteristic of CDSS, they are likely to retain applicability in less interventionist technological contexts. The framework is therefore not restricted to CDSS evaluation, but their centrality in contemporary AI-enabled healthcare makes them an analytically generative pilot domain.

### Concept and rationale

The term care optimizer does not describe all health technologies indiscriminately but rather denotes a normative ideal. A technology merits this designation only when it demonstrably supports the ethical aims of care—above all, patient wellbeing, relational integrity, and contextual responsiveness—through its mediating role. The term is aspirational and evaluative: it designates the role technologies should play. When a technology genuinely functions as a care optimizer, it does not simply improve patient outcomes in a narrow sense; it reshapes clinicians’ modes of knowing by recalibrating what appears salient, which forms of evidence are taken as authoritative, and how practical reasoning unfolds. Optimization here concerns the entire relational field—doctor, patient, and technology—rather than a unidirectional improvement of patient outcomes.

The dominant discourse of “optimization” is often associated with managerial logics of control, efficiency, and measurable performance. By contrast, we deliberately use “optimization” in a non-instrumental sense. It does not refer to maximizing a predefined outcome or a performance indicator, but to an ongoing, situated effort to sustain the relational conditions in which good care can emerge: attentiveness to vulnerability, responsiveness to changing needs, and trustful, dialogical engagement. Here, to “optimize” is to remain oriented toward these fragile, co-constructed practices under conditions of technological mediation, not to eliminate ambiguity or reduce care to a set of metrics. At its core, the care optimizer concept rests on the foundational aim of healthcare: to protect, restore, and enhance patient wellbeing. In conventional clinical interactions, the doctor acts as care provider and the patient as care receiver. Technologically mediated care introduces a third entity—neither provider nor receiver yet integral to the process. As a technological mediator, the care optimizer structures perception, guides decisions, enhances communication, personalizes interventions, and improves coordination and access. In doing so, it also participates in reorganizing medicine as a knowledge system, by privileging particular forms of evidence, diagnostic categories, and temporal horizons of care. This role is morally significant because technologies that enable, constrain, or organize care interactions can advance—or undermine—the relational and value-laden goals of medicine.

While this concept draws on Verbeek’s ideas of moral mediation and moralizing technology, it extends them in two ways. First, it frames technological mediation not only descriptively—how technologies shape perception and action—but normatively, orienting it toward the ethical aims of care: patient wellbeing, relational integrity, and contextual responsiveness. Second, it translates this normative orientation into a structured evaluative lens, identifying domains in which technology’s contribution to care can be assessed. In this sense, the care optimizer complements postphenomenology with a relationally oriented evaluative lens for judging whether technological influence in the clinic supports the moral aims of care.

Patient wellbeing—understood as promoting physical, psychological, and relational flourishing—anchors this framework. Relational integrity and contextual responsiveness are integral to this wellbeing, not alternatives to it. Instrumentalist models that treat technologies as inert tools fall short for two reasons. First, they assume functionality depends entirely on human intention, ignoring that technologies mediate perception and action, shaping moral and epistemic possibilities (Schatzberg 2018). Optimization here is not about doing more or faster; it is about structuring conditions for care to achieve its ethical telos. Second, instrumentalist framings overlook ethical implications: a system that defines relevant information, frames choices, and constrains action is never neutral. If medicine aspires to deliver *optimal* care, technologies must be assessed against that same ideal.

Heuristically, we can say that clinicians, patients, and technologies occupy different focal positions within the clinical relation—positions associated, respectively, with professional responsibility, embodied vulnerability, and mediating functions. Yet these roles are neither fixed essences nor stable points within a triangle. As the previous sections have shown, they are enacted and continually reconfigured through technological mediation: clinicians engage in knowing practices that are differently mediated, patients are interpreted within shifting moral and epistemic frames, and technologies shape, to varying degrees, what can be seen, expressed, and acted upon.

This model avoids anthropomorphizing technologies while recognizing their morally significant role. These roles are not ontologically prior to their relations; they emerge and shift through ongoing, technologically mediated practices of seeing, interpreting, and responding. Accordingly, optimizer status is contingent and context-dependent: the same technology may optimize care in one setting yet fail in another, depending on design, implementation, literacy, and sociotechnical conditions, and on how it redistributes epistemic authority and responsibility among the actors involved. For example, a CDSS supporting shared decision-making through transparent, patient-accessible visualizations can foster understanding and trust, whereas an opaque system may degrade care quality despite technical accuracy.

Thus, the care optimizer is both normative and evaluative. It articulates an ethical ideal for technology’s role while providing a critical lens for assessment. This underscores that technological participation in care must be grounded in patient wellbeing—the normative horizon of both clinical practice and mediation.

### Normative orientation and framework

Clarifying technologies’ ontological role as mediators is insufficient without normative orientation. Clinical contexts are inherently value-laden, and technological integration must be evaluated not merely on functional grounds but against explicit ethical criteria (Kelly et al. 2015). Positioning patient wellbeing at the center of evaluation provides this orientation. In this context, wellbeing is not a scalar, measurable outcome but an emergent quality of clinical life, produced through webs of attentiveness, trust, responsiveness, and mutual interpretation. We adopt a relational, context-sensitive understanding of wellbeing consistent with care ethics, recognizing its plural interpretations while treating it as the unifying normative horizon of clinical practice. In this framework, we understand care as a relational, interpretive practice oriented toward attentiveness, responsibility, and responsiveness within conditions of vulnerability. The role of CDSS, for instance, should not be limited to improving efficiency or diagnostic accuracy but aligned with medicine’s fundamental aim. Framing CDSS as care optimizers guided by wellbeing ensures that technological mediation reinforces, rather than erodes, the moral fabric of clinical practice.

This understanding of wellbeing aligns with care-ethical accounts, particularly Tronto’s phases of care: attentiveness, responsibility, competence, responsiveness, and solidarity (Tronto 2013). These phases describe not outcomes but ongoing practices of attending to needs, assuming and enacting responsibility, and responding appropriately to concrete others. To talk about “optimizing care” in our framework is therefore shorthand for sustaining and rearticulating these practices under conditions of technological mediation, not for maximizing a numerical score or index. Making wellbeing central also cannot be decoupled from the ways technologies reorganize what counts as valid knowledge and whose voices shape clinical reasoning: by privileging certain data, outcomes, or risk framings, CDSS participate in steering the epistemic direction of medicine as a knowledge system.

Within the clinic, CDSS do more than deliver data: they shape decision frames, distribute responsibility, and mediate patients’ experience of care (Onder 2025). Because technologies inevitably influence these relationships, their design and implementation must be guided by ethical ends rather than market-driven or purely functional imperatives.

Placing wellbeing at the normative core redefines technology’s role: from a passive support tool to an active enabler of moral practice. This demands that system architecture and workflows embed principles such as autonomy, transparency, and trustworthiness (Merchán Cruz et al. 2025; Braun et al. 2020). Making care optimization normatively meaningful requires concrete points of attention. Building on the conceptual foundation above, we identify five domains as relational sites where technologies may ethically co-shape care. These domains provide a multidimensional evaluative structure for assessing how technologies participate in care—and for guiding ethical design and governance. What we defend here, however, is that the increasing complexity of technologically mediated care necessitates a normative inclusivity of these criteria, ensuring that evaluations of integrated technology explicitly incorporate the moral and relational dimensions of care, rather than relying on purely functional or technical metrics.

### On the selection of the five domains

The five domains do not originate from health-services evaluation alone. Rather, they emerge at the intersection of three bodies of literature that repeatedly converge under conditions of technological mediation. First, quality-of-care scholarship, from Donabedian’s (1988) foundational structure-process-outcome model to later system frameworks (e.g., Committee on Quality of Health Care in America 2001; Arah et al. 2006), identifies safety, effectiveness, equity, and patient-centeredness as central dimensions of good care. Second, relational models of practice (Mead and Bower 2000; Mol 2008; Pols 2012) show that care is an interpretive and interactional process, not a sequence of isolated interventions. These perspectives reframe evaluative dimensions not as outcome variables but as relational accomplishments enacted through situated practices of interpretation, coordination, and responsiveness. Third, care-ethical accounts, especially Tronto’s phases of attentiveness, responsibility, competence, responsiveness, and solidarity, articulate the moral texture of these practices and provide normative vocabulary for evaluating their ethical adequacy (Tronto 1993, 2013). Unlike system-level quality-of-care frameworks such as the one by Committee on Quality of Health Care in America (2001)—which emphasize performance indicators like efficiency and timeliness—our domains foreground the quality of relationships and their ethical implications, as reshaped by technological mediation. In this sense, the proposed framework has an integrative character.

This integration operates through translation rather than synthesis. Quality-of-care frameworks identify clinically salient dimensions along which care succeeds or fails; relational and practice-based accounts reinterpret these dimensions as interactional and interpretive achievements; and care ethics articulates their moral significance. The five domains therefore mark recurring relational sites where technological mediation predictably reshapes perception, authority, communication, and responsibility, rather than representing categories derived from any single evaluative tradition.

The selection of these five domains emerged through recursive readings and analysis of the relevant literatures and internal deliberation among the authors, rather than through a systematic or geographically exhaustive review. We drew on recurring themes across health-services evaluation, design studies, and care-ethical and postphenomenological analyses, identifying points of convergence where technological mediation reconfigures clinical relationality. The domains thus reflect conceptual alignment across these traditions rather than a predefined taxonomy.

To select the domains, we applied three criteria. First, each domain had to be an area where technological mediation demonstrably alters underlying practices—for example, by shifting salience, redistributing interpretive authority, or reshaping communicative rhythms. Second, each domain needed to represent a relational configuration among patients, clinicians, technologies, and institutions rather than a discrete performance metric. Third, each domain had to possess both ethical salience (vulnerability, responsibility, trust, solidarity) and clinical salience (safety, quality, and governance).

These criteria clarify why familiar categories like timeliness or efficiency do not appear independently: they describe workflow properties rather than relational–ethical dynamics and are indirectly addressed within care practices and communication. Equity functions as a transversal value integrated across all domains rather than a distinct evaluative unit. Autonomy is similarly embedded; in technologically mediated contexts, it is enacted primarily through interpretive and communicative processes captured within decision-making.

Professional wellbeing forms a more nuanced case. Evidence on burnout, cognitive overload, and moral injury shows that clinician wellbeing shapes attentiveness, interpretive acuity, and communicative presence. Digital tools modulate these vulnerabilities by altering temporal rhythms, attention, and documentation demands. We therefore incorporate clinician wellbeing indirectly within patient-centeredness, decision-making, and care practices. Making it an independent domain would redirect the framework’s normative focus away from patient wellbeing—the ethical telos of medicine—while omitting it would obscure a key relational condition for good care.

A further justification comes from the correspondence between the domains and Tronto’s phases of care: attentiveness (patient-centeredness), responsibility (decision-making), competence (care quality), responsiveness (access and communication), and solidarity (care practices). This mapping shows that the domains are not arbitrary inventions but grounded in an established account of ethically adequate care.

Within this horizon, patient wellbeing—understood in the sense articulated by the German Ethics Council as enabling self-determination and the conditions for meaningful care—is a relational achievement rather than a measurable endpoint (Deutscher Ethikrat 2016). The five domains identify the principal relational mechanisms through which wellbeing is cultivated or undermined when technologies participate in the clinical encounter.

Although analytically distinguishable, the domains overlap by design: in practice, attentiveness, communication, reasoning, and institutional support unfold together. Distinguishing them allows evaluators to locate how specific forms of technological mediation exert ethical and epistemic influence without implying that care decomposes into modular components.

Finally, the framework is intentionally dynamic. Technological mediation continually reshapes what becomes salient or actionable, so the domains should not be seen as a closed taxonomy. New technologies may render additional domains relevant; existing ones may subdivide or converge. What endures is a methodological commitment—demonstrable mediation, relational orientation, and ethical–clinical salience—which positions the framework as a living program for inquiry and governance. Accordingly, the application and interpretation of the five domains justified above should be case-based and oriented toward concrete ethical questions rather than toward a uniform evaluative scheme. The framework is intended to guide context-sensitive ethical reasoning, rather than to prescribe a single mode of application or interpretation.

### The five domains of care optimization

Viewed through postphenomenology, the five domains are sites of mediation where technologies reconfigure perception, communication, and shared meanings. Care ethics, in turn, interprets these sites as practices of attentiveness, responsiveness, and relational integrity. Thus, the five domains do not operate as managerial categories or performance indicators, but as ethically charged relational zones in which the moral texture of technologically mediated care is enacted.

The following five domains—care quality, patient-centeredness, decision-making, access and communication, and care practices—serve as normative lenses for determining whether, in practice, a technology can legitimately be called a *care optimizer*. These are not a checklist of inherent properties; rather, they are evaluative criteria that examine how technological mediation aligns with the ethical and clinical aims of medicine. Just as “optimal care” in clinical practice is never assumed but achieved through deliberate alignment with standards, the same applies to technology: only when mediation advances care in these domains can it optimize care.

These categories are neither exhaustive nor mutually exclusive; they often overlap and influence each other. Together, they form a comprehensive schema for evaluating the ethical and relational adequacy of health technologies—particularly CDSS—in contemporary clinical environments.

#### Care quality

Care quality is a central concern in both medical ethics and health policy. Campbell et al. (2000) identify two primary dimensions of healthcare quality: accessibility and effectiveness. While access is addressed in Sect. 4.3.4, here we focus on effectiveness—defined as the ability of care interventions to improve health outcomes, reduce harm, and maintain reliability.

Evidence indicates that CDSS can enhance clinical effectiveness by reducing medication errors, standardizing adherence to evidence-based guidelines, and improving diagnostic accuracy (Bright et al. 2012; Kawamoto et al. 2005). Their impact is particularly significant in chronic disease management, where continuous monitoring and timely interventions prevent complications and support patient wellbeing.

The integration of real-time analytics from EHRs enables personalized treatment plans reflecting both clinical evidence and individual patient data (Batko and Ślęzak 2022). Such integration can facilitate earlier detection, more accurate prognoses, and lower adverse event rates.

However, improvement in care quality is not guaranteed. Poorly designed or inadequately validated systems may introduce risks, including overreliance on algorithmic output, deskilling of clinicians, or automation of errors (Gaitanou et al. 2014; Duran 2021). Technical performance alone does not justify the “optimizer” label; ethical vigilance is essential to ensure systems deliver safe, context-sensitive care without undermining professional judgment or relational integrity.

From a postphenomenological perspective, care quality is never purely technical: it is co-defined by epistemic values embedded in the system, such as which outcomes are prioritized, which risks are made visible, and which forms of evidence are sidelined. CDSS that foreground guideline adherence or specific outcome metrics, for instance, indirectly reshape what counts as “good medicine” and how clinicians understand their professional excellence. In this way, technologies that purport to optimize quality actively participate in reconfiguring medicine as a knowledge system, not merely in improving local performance. Thus, care quality becomes both a clinical and normative benchmark: a technology functions as a care optimizer only when it supports better outcomes without compromising trust, safety, or human-centered practice.

#### Patient-centeredness

Patient-centered care frames the patient not as a constellation of symptoms but as a situated person embedded in a lived context of values, relationships, and preferences. Emerging against paternalistic models, this paradigm emphasizes autonomy, empathetic communication, and shared decision-making (Balint 1969; Stewart et al. 1995). Contemporary discussions of patient-centeredness increasingly treat it not as a singular procedural requirement but as a multi-layered orientation spanning communication, engagement, and organizational responsiveness. Conceptual frameworks in healthcare research emphasize domains such as respectful interaction, patient participation in care management, and integration of care processes across settings (Santana et al. 2018; Wasim et al. 2023). Within clinical practice, patient-centered communication has been identified as a core mechanism through which dignity, shared interpretation, and collaborative decision-making are enacted, inviting patients and families to actively negotiate their care trajectories (Kwame and Petrucka 2021). Moreover, recent developments linking patient-centered care to the rise of personalized and precision medicine illustrate how responsiveness to individual contexts is becoming structurally embedded within contemporary healthcare systems (Stefanicka-Wojtas and Kurpas 2021). These developments support interpreting patient-centeredness here not as a measurable endpoint, but as a relational condition shaping technologically mediated encounters.

Technologies can reinforce this ideal when ethically designed. CDSS that integrate patient preferences, historical health data, and personal risk profiles can personalize recommendations in meaningful ways. Research confirms that algorithms incorporating patient preferences significantly influence treatment decisions, promoting value-concordant care (Fusiak et al. 2025). For example, a decision-support system that flags treatment options conflicting with prior care goals actively fosters autonomy.

Patient-facing tools—such as interactive dashboards and visual explanation systems—enhance comprehension and participation, improving informed consent. Lo and Parham (2010) argue that such tools strengthen trust and transparency by enabling patients to actively engage in their care. Patient-centeredness is therefore best understood as a relational interpretive practice rather than a simple tailoring of outputs. Technologies may amplify this practice by making room for patients’ narratives and preferences, but they can also recentre only those subjectivities that are data-rich, digitally literate, or easily codified, thereby marginalizing others. In shaping whose experiences become legible and actionable, CDSS and related tools redistribute epistemic authority between patients, clinicians, and data infrastructures. We retain the term ‘patient-centeredness’ given its established role in clinical ethics, while acknowledging that person-centeredness provides a broader lens that emphasizes the individual’s lifeworld beyond the clinical encounter.

Nevertheless, tensions persist. Algorithms often operate on standardized datasets, risking erosion of individuality. Personalization may become superficial—limited to demographic tailoring rather than contextual understanding. Furthermore, automated prompts or rigid templates may inadvertently reduce clinician flexibility, diminishing dialogical engagement.

To qualify as a care optimizer, technology must extend—not replace—the interpretive practices central to patient-centered care. This requires design strategies attentive to cultural, linguistic, and cognitive accessibility, as well as continuous feedback loops that account for diverse patient experiences.

#### Decision-making

Clinical decision-making is both cognitively and ethically demanding, requiring navigation of uncertainty, interpretation of complex data, and reconciliation of competing values. The Shared Decision-Making (SDM) model has become an ethical standard for promoting autonomy and collaboration, especially when multiple reasonable options exist (Elwyn et al. 2012).

Technologies can facilitate SDM through decision aids that present risks, benefits, and options in accessible formats—visualizations, interactive simulations, and outcome calculators. For example, Paling (2003) demonstrates how visual tools improve risk comprehension, enabling informed patient choices.

AI-enabled CDSS further extend these capabilities by generating personalized predictions, such as survival probabilities or treatment-specific risk profiles. This data-driven personalization can enrich deliberation when combined with patient narratives and clinician expertise. In many cases, AI-enabled decision support also shifts the form of clinical reasoning itself—from narrative, analogical, or pathophysiological inference toward probabilistic, predictive, and pattern-based evaluation. This does not simply add more information; it nudges clinicians toward particular epistemic stances, such as risk management or population-level optimization, which may sit uneasily with individualized, relational understandings of care. The care optimizer framework therefore asks not only whether decisions are technically improved, but how technologies reconfigure who the knower is and what kinds of knowing medicine comes to privilege.

However, ethical challenges remain. Overreliance on algorithmic outputs risks diminishing clinicians’ critical judgment and the collaborative character of SDM. Opacity compounds this problem: if patients—and often clinicians—cannot understand how recommendations are generated, trust and meaningful consent suffer (Lorenzini et al. 2023). Health literacy and digital literacy disparities exacerbate inequities, leaving vulnerable groups at a disadvantage (Onder 2025).

For technologies to optimize decision-making, they must enhance transparency, support dialogue, and maintain human interpretive agency. Rather than displacing the clinician-patient conversation, they should scaffold it—providing clarity without foreclosing deliberation.

#### Access and communication

Access to healthcare is a foundational determinant of wellbeing. Digital innovations such as telehealth platforms, mobile health applications, and EHR-integrated portals have been celebrated for expanding care beyond physical clinics, improving continuity, and reducing geographical barriers (Blumenthal 2010; Granström et al. 2020). Telehealth particularly benefits rural and underserved populations by enabling specialist consultations without burdensome travel. Asynchronous tools—secure messaging or remote monitoring dashboards—allow timely interaction, increasing flexibility for both patients and clinicians.

Technologies also strengthen intra-professional communication: EHR systems facilitate efficient information sharing, while digital coordination platforms align multidisciplinary care (Weiner et al. 2012).

However, access is not merely a question of availability; it implicates equity and usability. The digital divide persists: disparities in internet access, device ownership, and digital literacy disproportionately affect older adults, marginalized communities, and those with low socioeconomic status (Yang et al. 2024). Privacy and data security concerns further deter patient engagement (Alhammad et al. 2024). Ethical expectations of confidentiality in digitally mediated care environments are increasingly shaped by relational configurations of cyberspatial proximity and distance, highlighting the normative significance of spatial mediation in healthcare technologies (Onder and Deniz 2025).

Moreover, an increased reliance on digital communication can foster depersonalization (Akingbola et al. 2024). The reduction of embodied encounters risks eroding empathy and trust—values integral to therapeutic relationships (Powell 2024; Stieger et al. 2023).

Access and communication are also shaped by institutional networks, infrastructural choices, and platform designs that filter which voices can enter clinical conversations and whose data “matters.” Patient portals, triage systems, and messaging platforms prioritize some forms of expression (structured forms, numerical scores, short messages) over others (long narratives, non-verbal cues), thereby mediating whose concerns are heard and how they are interpreted. In this respect, technologies of access and communication help rearticulate the boundaries of the clinical community and the distribution of epistemic authority within it. For technologies to optimize access and communication, they must ensure inclusive design, privacy protection, and the preservation of relational depth in digitally mediated environments.

#### Care practices and coordination

Technologies reshape not only diagnostic or communicative dimensions of care but also its organizational foundations. CDSS and related tools serve as infrastructure for workflow optimization, resource management, and interdisciplinary coordination.

Automation of routine tasks—appointment scheduling, prescription refills, or structured documentation—reduces administrative burdens, allowing clinicians to reallocate time toward relational and diagnostic care (Kuperman et al. 2007). This alleviation of cognitive and logistical strain can mitigate burnout and enhance job satisfaction.

Predictive analytics extend optimization into the temporal dimension of care. AI-driven models trained on longitudinal health data can anticipate clinical deterioration, identify high-risk patients, and recommend proactive interventions. Evidence shows that early warning systems reduce in-hospital and 30-day mortality while shortening hospital stays (Yuan et al. 2025). Systems implementing predictive analytics report cost reductions of up to 25% and 15–20% lower readmission rates (Hossain et al. 2024).

Coordination is equally critical. Digital platforms integrate distributed actors—clinicians, nurses, specialists, and caregivers—facilitating synchronized, holistic responses for patients with complex needs. Yet these benefits depend on interoperability, usability, and organizational alignment. Absent these conditions, technologies risk fragmenting workflows or introducing inefficiencies.

Ethical governance must accompany infrastructural innovation. Following the embedded ethics model (Coeckelbergh and Stahl 2016), system design and deployment should foreground empathy, attentiveness, and human presence—even when mediated digitally. Care optimization, therefore, involves not only logistical gains but moral quality enhancement across the institutional landscape.

These infrastructures do more than streamline workflows: they weave dense networks of epistemic interdependence in which protocols, algorithms, and institutional routines co-produce what counts as timely, appropriate, or even thinkable care. Over time, such systems influence training curricula, guideline development, and organizational priorities, thereby steering the long-term direction of medicine as a knowledge system. A technology counts as a care optimizer in this domain only when these reorganizations of practice and knowledge remain accountable to the relational and ethical ideals of care, rather than to efficiency or cost savings alone.

Across these five domains—care quality, patient-centeredness, decision-making, access and communication, and care practices—emerges a multidimensional account of care optimization. These domains are evaluative rather than descriptive: technologies do not automatically fulfill them but must be deliberately aligned with medicine’s ethical telos. The next section examines the broader normative implications of this framework for clinical practice, design, and governance.

## Discussion

The concept of care optimization seeks to contribute to ongoing debates on technological mediation by suggesting a normative orientation grounded in patient wellbeing. While contemporary assessments of digital health tools often prioritize technical metrics—accuracy, efficiency, and cost-effectiveness (Bright et al., 2012; Kawamoto et al. 2005)—these measures fail to capture the moral telos of medicine: the protection and promotion of patient wellbeing. Care ethics has long insisted that good care entails attentiveness, relational integrity, and responsiveness to context (Tronto 1993), yet these values remain insufficiently integrated into evaluations of technological systems. Conversely, existing frameworks in clinical ethics articulate normative ideals such as patient-centeredness and shared decision-making (Elwyn et al. 2012; Epstein and Street 2011) but rarely consider how technologies materially co-constitute these practices. In the remainder of this discussion, we therefore concentrate on the conceptual contribution of the framework, the tensions it exposes, and the limits that define its scope, rather than reiterating its descriptive foundations.

Postphenomenology disrupts instrumentalist assumptions by framing technologies not as neutral tools but as mediators that shape perception, action, and meaning (Ihde 1990; Verbeek 2005, 2011). However, this analysis has remained largely descriptive, leaving open the question of what ethical ends such mediation should serve (Pols 2012; Rosenberger and Verbeek 2015). The care optimizer framework seeks to address this gap by linking descriptive and conceptual insights from postphenomenology with a proposed normative structure anchored in patient wellbeing, offering a conceptual approach for reflecting on the ethical orientation of technological mediation.

Central to this approach is the consolidation of five normative domains—care quality, patient-centeredness, decision-making, access and communication, and care practices—into a unified evaluative schema. These domains have been invoked in fragmented ways across health policy and design literature, but our framework attempts to situate them within a more coherent ethical orientation grounded in wellbeing. This structure is intended to serve two functions that may help clarify the normative role of technology. First, it clarifies that these categories are not managerial targets or procedural conveniences; they are relational sites for assessing whether technologies advance or erode the foundational goals of care. Second, it operationalizes wellbeing without reducing it to abstract principle, offering a set of tentative evaluative criteria that could help inform clinical assessment, design processes, and policy development.

The five domains can be briefly revisited here not to restate their content, but to clarify how they structure ethical interpretation of technological mediation in practice. Care quality, for example, must go beyond technical performance to include safe, context-sensitive interventions that uphold professional judgment (Bright et al. 2012; Gaitanou et al. 2014). Patient-centeredness requires technologies that respect individuality, incorporating preferences without reducing them to algorithmic templates (Balint 1969; Stewart et al. 1995; Fusiak et al. 2025). Decision-making should not only be data-driven but deliberative, supporting transparency and enabling relational dialogue while mitigating the risks of overreliance on opaque systems (Elwyn et al. 2012; Lorenzini et al. 2023). Access and communication demand inclusivity, addressing digital divides and preserving the relational depth of care amid telehealth proliferation (Blumenthal 2010; Yang et al. 2024; Powell et al. 2024). Finally, care practices and coordination underscore the need for infrastructural design that facilitates not just efficiency but the moral quality of institutional workflows, embedding ethics into predictive analytics and automation (Kuperman et al. 2007; Hossain et al. 2024).

By grounding these domains in patient wellbeing, our framework resists the reduction of “optimization” to operational efficiency—a risk amplified by market pressures and managerial rationalities (Blumenthal 2010). Optimization here is redefined as an ethically charged orientation: structuring technological mediation to achieve care that is relationally responsive, contextually appropriate, and clinically sound. This reframing draws on postphenomenology and tentatively extends its descriptive insights into a more normatively oriented approach, answering the question of not only *how* technologies mediate but *how they should mediate* to sustain medicine’s moral horizon.

Nonetheless, adopting this framework brings several structural tensions into focus that delimit its normative reach. Responsibility distribution remains a persistent challenge: as CDSS shape clinical reasoning by framing risks and recommendations, they influence decisions in ways that complicate accountability (Verbeek 2011; Funer et al. 2024b). Clinicians often experience uncertainty about liability when algorithmic mediation intervenes in judgment (Lorenzini et al. 2023), highlighting the importance of explainability and appropriate governance structures that maintain human interpretive agency (Friedrich et al. 2022). Depersonalization poses another risk: while digital platforms enhance access, they may fragment interactional space, reducing opportunities for empathetic engagement (Scott and Purves 1996; Powell 2024). Similarly, digital inequities threaten to restrict optimization benefits to privileged populations, reinforcing structural disparities (Yang et al. 2024). Finally, the rhetoric of optimization can be co-opted by economic imperatives, collapsing moral aims into performance metrics unless normative benchmarks—such as the ones proposed here—are explicitly embedded in evaluative and regulatory frameworks. Two objections merit clarification. First, optimization rhetoric may appear technocratic; we resist this by anchoring optimization in ethical telos rather than efficiency metrics. Second, referring to technologies as moral actors does not confer agency or liability but highlights their role in shaping conditions for action, while ultimate responsibility remains human.

At the same time, several limitations clarify the intended scope of the framework. The care optimizer concept is deliberately relational and interpretive rather than procedural, and it does not provide a standardized metric or evaluative algorithm. Its application therefore depends on contextual judgment within specific clinical environments. Moreover, patient wellbeing remains plural and contested, requiring situated interpretation rather than universal operationalization. Technological mediation itself is multistable: systems that enhance relational attentiveness or coordination in one setting may introduce opacity, deskilling, or depersonalization in another. Finally, despite our non-instrumentalist use of the term, the language of “optimization” remains normatively vulnerable to managerial reinterpretation, underscoring the need to continually anchor evaluation in relational and ethical considerations rather than performance logics.

These tensions carry important practical implications, though their resolution may vary across clinical settings. Clinicians must cultivate ethical and technological literacy, recognizing technologies as co-actors that shape responsibility, autonomy, and trust within the clinical encounter (James et al. 2023). Training should incorporate reflection on mediation effects alongside functional proficiency. Evaluative metrics must evolve to include not only accuracy and error reduction but also relational indicators such as communication quality, patient trust, and shared decision-making outcomes (Funer et al. 2024a). On the design side, embedding ethical reflection through Responsible Research and Innovation approaches (Stahl and Coeckelbergh 2016; Brey 2012) is essential. This entails integrating explainability, cultural accessibility, and equity considerations into system architecture (Lo and Parham 2010; Merchán-Cruz et al. 2025; Braun et al. 2020). Governance frameworks such as the EU AI Act (Regulation 2024/1689) should expand beyond technical compliance to incorporate wellbeing-centered benchmarks within health technology assessment processes.

Despite these challenges, the framework offers a compelling advance: it aims to anchor technological evaluation within a more coherent ethical vision, extending postphenomenology into normative territory while retaining its sensitivity to the materiality of mediation. By articulating patient wellbeing as the basis, five categories as relational and moral practices, and care optimization as a normative lens, we move toward a richer account of what it means for technologies to serve—not distort—the moral fabric of healthcare. In this sense, care optimization is not a static model but a dynamic program for inquiry and governance, guiding the integration of digital health technologies toward an ethically meaningful future.

To clarify how responsibility and moral relevance are distributed within this framework—especially in discussions of mediation and accountability—a terminological distinction is required. In this article, we use actor to denote any entity—human or technological—that shapes the conditions under which action unfolds, whereas agent refers only to entities capable of intentional, accountable action. Referring to digital systems as “moral actors” does not attribute agency, intentionality, or liability to them. Rather, it emphasizes that technologies shape the conditions under which human agency unfolds. They mediate attention, frame decisions, and structure communication, thereby participating in the moral composition of clinical action without becoming moral subjects. This distinction preserves human accountability while acknowledging the normative relevance of technological mediation within clinical practice. While patient wellbeing constitutes the normative anchor of the care optimizer framework, it cannot be sustained independently of the conditions under which care is provided. Patient wellbeing is relationally achieved, and one of its central enabling conditions is the wellbeing of clinicians themselves. Technological mediation therefore affects not only patients but also clinicians, whose attentiveness, judgment, and moral resilience shape the quality of care that can be delivered.

Clinician wellbeing warrants explicit recognition as part of the relational ecology of care optimization. Burnout, moral injury, and cognitive overload affect attentiveness, interpretive acuity, and communicative presence, all of which directly shape patient wellbeing. While clinician wellbeing is not introduced as a standalone domain, it remains embedded across patient-centeredness, decision-making, and care practices, functioning as a background condition for the ethical orientation of technologically mediated care.

These clarifications collectively sharpen the boundaries of the framework and foreground the constraints under which its normative claims should be interpreted. Against this background, two integrative conclusions can be drawn from the care optimizer framework, which also point toward broader implications for its application and evaluation.

1. Applicability is necessarily context- and discipline-sensitive. A technology may facilitate optimal coordination in surgical or emergency settings yet compromise relational trust in psychiatry or palliative care. Whether a system can be considered a care optimizer depends on how its mediating effects interact with the clinical, sociotechnical, and epistemic demands of a particular field.
2. Evaluation must remain responsive to individual patient experience. Wellbeing cannot be imposed through fixed criteria; it emerges in lived encounters shaped by values, vulnerabilities, and differing thresholds of digital literacy. A system that empowers one patient may overwhelm another. For this reason, care optimization requires responsiveness at both institutional and interpersonal levels, ensuring that technologies support—not displace—the plural ways in which patients experience good care.

Together, these clarifications reinforce the framework’s central claim: technologies can be evaluated as care optimizers only when their mediating effects align with the ethical commitments of care—contextually, relationally, and personally.

## Conclusion

The integration of artificial intelligence and digital technologies into healthcare will continue to reshape not only the efficiency and organization of clinical practice but also its moral and relational constitution. Throughout this article, we have argued that these technologies cannot be understood as neutral instruments; they mediate how care is perceived, enacted, and experienced. By introducing the concept of the care optimizer, we offered a normative framework that links postphenomenological insights with the ethical telos of medicine, positioning patient wellbeing as the central orientation for evaluating technologically mediated care.

The five domains identified, care quality, patient-centeredness, decision-making, access and communication, and care practices, are best understood as relational sites rather than technical metrics. They articulate how technologies may support, redirect, or undermine the conditions under which good care is possible. By grounding these domains in wellbeing and demonstrating how their significance varies across clinical contexts and individual patient experiences, the framework invites a more situated, ethically responsive evaluation of digital health technologies. Future work should refine these criteria through empirical research and participatory methodologies, ensuring that innovations in AI and digital systems remain aligned with medicine’s foundational commitment to relationally attentive, contextually sensitive, and ethically robust care.
